# Insights into Mad2 Regulation in the Spindle Checkpoint Revealed by the Crystal Structure of the Symmetric Mad2 Dimer 

**DOI:** 10.1371/journal.pbio.0060050

**Published:** 2008-03-04

**Authors:** Maojun Yang, Bing Li, Chyong-Jy Liu, Diana R Tomchick, Mischa Machius, Josep Rizo, Hongtao Yu, Xuelian Luo

**Affiliations:** 1 Department of Pharmacology, The University of Texas Southwestern Medical Center, Dallas, Texas, United States of America; 2 Department of Biochemistry, The University of Texas Southwestern Medical Center, Dallas, Texas, United States of America; Princeton University, United States of America

## Abstract

In response to misaligned sister chromatids during mitosis, the spindle checkpoint protein Mad2 inhibits the anaphase-promoting complex or cyclosome (APC/C) through binding to its mitotic activator Cdc20, thus delaying anaphase onset. Mad1, an upstream regulator of Mad2, forms a tight core complex with Mad2 and facilitates Mad2 binding to Cdc20. In the absence of its binding proteins, free Mad2 has two natively folded conformers, termed N1-Mad2/open-Mad2 (O-Mad2) and N2-Mad2/closed Mad2 (C-Mad2), with C-Mad2 being more active in APC/C^Cdc20^ inhibition. Here, we show that whereas O-Mad2 is monomeric, C-Mad2 forms either symmetric C-Mad2–C-Mad2 (C–C) or asymmetric O-Mad2–C-Mad2 (O–C) dimers. We also report the crystal structure of the symmetric C–C Mad2 dimer, revealing the basis for the ability of unliganded C-Mad2, but not O-Mad2 or liganded C-Mad2, to form symmetric dimers. A Mad2 mutant that predominantly forms the C–C dimer is functional in vitro and in living cells. Finally, the Mad1–Mad2 core complex facilitates the conversion of O-Mad2 to C-Mad2 in vitro. Collectively, our results establish the existence of a symmetric Mad2 dimer and provide insights into Mad1-assisted conformational activation of Mad2 in the spindle checkpoint.

## Author Summary

Chromosome missegregation during mitosis results in the gain or loss of chromosomes in the next generation of cells and can contribute to birth defects or cancer. A cellular surveillance system called the spindle checkpoint ensures that accurate chromosome segregation occurs by inhibiting the activity of the anaphase-promoting complex or cyclosome (APC/C) until all sister chromatids have achieved proper attachment to the mitotic spindle. The spindle checkpoint protein Mad2 binds to Cdc20, an activator of APC/C, and inhibits the complex. The Mad2 protein can adopt either an open or closed conformation. The conformational switch in Mad2 is critical for Cdc20 binding and APC/C inhibition, and is regulated by the protein Mad1. We report the crystal structure of the symmetric Mad2 dimer, which is made up of two closed monomers, and is active in APC/C-Cdc20 inhibition. Mad1 seems to facilitate the open–closed conformational switch of Mad2, and we present a unified model to explain Mad1-assisted Mad2 activation in the spindle checkpoint.

## Abstract 3

Analysis of the crystal structure and biochemical activity of the symmetric closed Mad2 dimer support a unified model for Mad1-assisted conformational activation of Mad2 in the spindle checkpoint.

## Introduction

At the metaphase–anaphase transition, a multisubunit ubiquitin ligase called the anaphase-promoting complex or cyclosome (APC/C) in complex with its mitosis-specific activator Cdc20 mediates the ubiquitination of securin and cyclin B [1,2]. Degradation of securin and cyclin B activates separase, which cleaves the Scc1 subunit of cohesin and triggers sister-chromatid separation [1,2]. Premature sister-chromatid separation leads to aneuploidy, which contributes to cancer progression [3,4]. In response to the existence of sister chromatids that lack attachment of spindle microtubules at their kinetochores, a cell-cycle surveillance system called the spindle checkpoint inhibits APC/C^Cdc20^ through multiple mechanisms, stabilizes securin and cyclin B, and delays the onset of anaphase [2,3,5]. The spindle checkpoint protein Mad2 binds directly to Cdc20 in mitosis and is essential for checkpoint-dependent inhibition of APC/C [6–8]. Binding of Mad2 to Cdc20 requires Mad1, an upstream regulator of Mad2 that binds to Mad2 throughout the cell cycle [9–11]. Both Mad1 and Cdc20 contain similar short peptide motifs that mediate Mad2 binding [11]. Either inactivation or hyperactivation of Mad2 promotes tumorigenesis in mice [12,13], highlighting the importance of proper Mad2 regulation in vivo. A series of biochemical, cell biological, and structural studies has established that Mad2 is a highly unusual two-state protein and that the Mad1-assisted conformational switch between these two states is central to Mad2 regulation [5,14].

In an early study, Fang, et al. [8] showed that recombinant purified Mad2 has two natively folded conformers, a monomer and a dimer, in the absence of ligand binding or covalent modification. The Mad2 dimer can form tetramers at high concentrations. The Mad2 dimer, but not the monomer, is active in APC/C inhibition in *Xenopus* egg extracts. Furthermore, the Mad2 monomer blocks the function of the Mad2 dimer in a dominant-negative manner. Structural studies were subsequently carried out to explain this striking two-state behavior of Mad2. The structures of the Mad2 monomer and Mad2 in complex with either Mad1 or an unnatural peptide ligand called Mad2-binding peptide 1 (MBP1) that mimics the Mad2-binding motifs of Mad1 or Cdc20 were determined [11,15,16]. These structures revealed that the Mad2 monomer has a globular domain and a flexible C-terminal tail. A Mad2 mutant with its C-terminal tail deleted (Mad2^ΔC^) is an open Mad2 (O-Mad2) monomer, is incapable of binding to Cdc20, and inhibits the activity of wild-type Mad2 in a dominant-negative manner. Mad2 undergoes a dramatic conformational change upon ligand binding. The peptide ligands are trapped by the C-terminal region of Mad2 in a manner similar to the way that passengers are restrained by the seat belts in automobiles.

The Mad2 point mutant, Mad2^R133A^, has two distinct monomeric conformers in the absence of ligands, which allowed us to determine the structure of both natively folded conformers of Mad2^R133A^, termed N1-Mad2/open Mad2 (hereafter referred to as O-Mad2) and N2-Mad2/closed Mad2 (C-Mad2), by nuclear magnetic resonance (NMR) spectroscopy [17]. (We initially named these two conformers N1-Mad2 and N2-Mad2. To avoid confusion, however, we have decided to adopt the nomenclature of De Antoni et al. [18].) The structure of unliganded C-Mad2 closely resembles that of Mad1- or Cdc20-bound C-Mad2 except that the ligand-binding site is vacant. O-Mad2 can spontaneously convert to C-Mad2 with slow kinetics (*t*_1/2_ = 9 h at 30 °C) [17]. Furthermore, cytosolic Mad2 in human cells is an O-Mad2 monomer [17]. Monomeric C-Mad2^R133A^, but not O-Mad2^R133A^, is active in APC/C^Cdc20^ inhibition. In addition, O-Mad2 and C-Mad2 can form an asymmetric O-Mad2–C-Mad2 (O–C) dimer that is less active in APC/C^Cdc20^ inhibition [17], explaining why Mad2^ΔC^ (which only adopts the O-Mad2 conformation) can block the activity of wild-type Mad2 in a dominant-negative manner. Finally, Mad1 facilitates the conversion of O-Mad2 to C-Mad2 in vitro [17]. Mad2 is targeted to unattached kinetochores by Mad1 and turns over rapidly at the kinetochores as revealed by fluorescence recovery after photobleaching (FRAP) studies [9,19–21]. These studies suggest that Mad1 activates Mad2 at kinetochores by facilitating the structural conversion of O-Mad2 to C-Mad2.

More recent FRAP studies revealed that only about 50% of kinetochore-bound Mad2 undergoes fast exchange with its cytosolic pool [22], suggesting that there is a stably bound pool of Mad2 at the kinetochores. Musacchio and coworkers then showed that this stably kinetochore-bound pool of Mad2 forms a tight complex with Mad1 and adopts the C-Mad2 conformation [16,18]. The Mad1–Mad2 core complex recruits cytosolic O-Mad2 to kinetochores through asymmetric O–C Mad2 dimerization.

**Figure 1:**
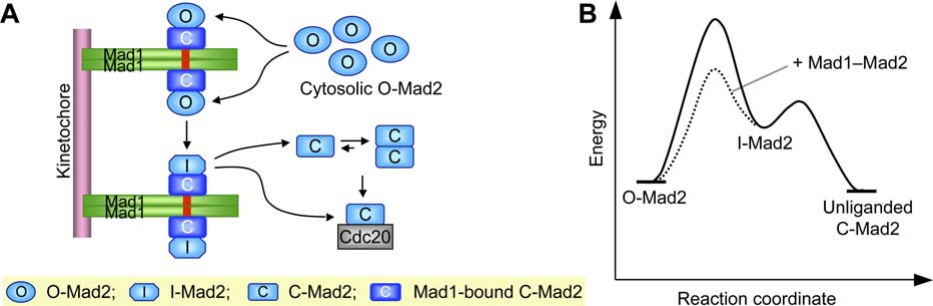
Model for Mad1-Assisted Mad2 Activation during Checkpoint Signaling (A) A model for the conformational activation of Mad2. The symbols used for different Mad2 conformers are shown in the yellow box. The Mad2-binding motif of Mad1 is colored red. (B) Energy diagram for the Mad2 conversion. In the absence of Mad1 or Cdc20, purified O-Mad2 spontaneously converts into unliganded C-Mad2, because O-Mad2 is at a slightly higher energy state relative to unliganded C-Mad2. We postulate that the O–C Mad2 conversion proceeds via an undefined intermediate state of Mad2, termed I-Mad2. The energetic barrier between C-Mad2 and I-Mad2 may be lower than that between O-Mad2 and I-Mad2. Thus, C-Mad2 can reach the I-Mad2 conformation more easily than O-Mad2, explaining why C-Mad2 is more active in APC/C^Cdc20^ inhibition. The Mad1–Mad2 core complex facilitates the O–C Mad2 conversion by lowering the energetic barrier between O-Mad2 and I-Mad2.

All available data thus support the following main framework to explain the mechanism by which Mad1 assists the binding of Mad2 to Cdc20 (Figure 1) [14,17,18,23–26]. In this model, Mad2 has two distinct conformations of roughly equal free energy: a latent O-Mad2 and an active C-Mad2. The Mad1–Mad2 core complex recruits another copy of cytosolic O-Mad2 to kinetochore through O–C Mad2 dimerization. O-Mad2 bound to the Mad1–Mad2 core complex undergoes a conformational change to adopt a short-lived, high-energy intermediate conformation (I-Mad2). (I-Mad2 was previously referred to as O*-Mad2. To avoid confusion, we will use the unified nomenclature described in [24,25].) I-Mad2 can be directly passed onto Cdc20 from the Mad1–Mad2 core complex. Alternatively, at least a fraction of I-Mad2 converts to unliganded C-Mad2, which dissociates from Mad1. Because Mad1 is a homodimer, two C-Mad2 molecules dissociated from Mad1 are expected to form a symmetric C-Mad2–C-Mad2 (C–C) Mad2 dimer. These unliganded C-Mad2 species are more active for Cdc20 binding and APC/C inhibition. Chemical shift perturbation experiments had initially suggested that, upon binding to C-Mad2, O-Mad2 undergoes a large conformational change to become I-Mad2 [23]. The structure of the asymmetric O-Mad2–C-Mad2 dimer has, however, revealed that O-Mad2 bound to C-Mad2 has virtually the same conformation as does free O-Mad2 [25]. Thus, I-Mad2 is not the stable conformation of O-Mad2 bound to C-Mad2, but rather a high-energy state with a finite lifetime. The existence and nature of I-Mad2 remain to be established.

In this study, we performed systematic mutagenesis studies of human Mad2 and obtained Mad2 mutants that preferably adopt the closed conformation. We determined the crystal structure of one such mutant, Mad2^L13A^, demonstrating unequivocally that C-Mad2 can form a symmetric C–C dimer in vitro. Using NMR spectroscopy, we showed that the wild-type Mad2 can form both an asymmetric O–C dimer and a symmetric C–C dimer. Mad2^L13A^, which predominantly exists as the symmetric C–C Mad2 dimer, is functional in cells and is active in APC/C^Cdc20^ inhibition in vitro. Finally, the Mad1–Mad2 core complex enhances the conversion of O-Mad2 to C-Mad2. These findings provide further mechanistic insights into the conformational activation of Mad2 by Mad1 in the spindle checkpoint.

## Results/Discussion

### Identification of Conformation-Specific Mad2 Mutants

We have previously shown that Mad2^R133A^ forms monomeric O-Mad2 and C-Mad2 conformers that interconvert with slow kinetics [17]. The monomeric open and closed conformers of Mad2^R133A^ can be separated by anion exchange chromatography at 4 °C. O-Mad2 elutes at 150 mM salt, whereas C-Mad2 elutes at 260 mM salt. Inspection of their surface electrostatic potentials reveals that C-Mad2 contains a contiguous, negatively charged patch centered around β6 that is absent in O-Mad2 because β6 is largely buried by β7 and β8 (Figure S1). The presence of this negatively charged patch provides a possible explanation for the tighter association of C-Mad2 with the positively charged resin of the anion exchange column.

**Figure 2:**
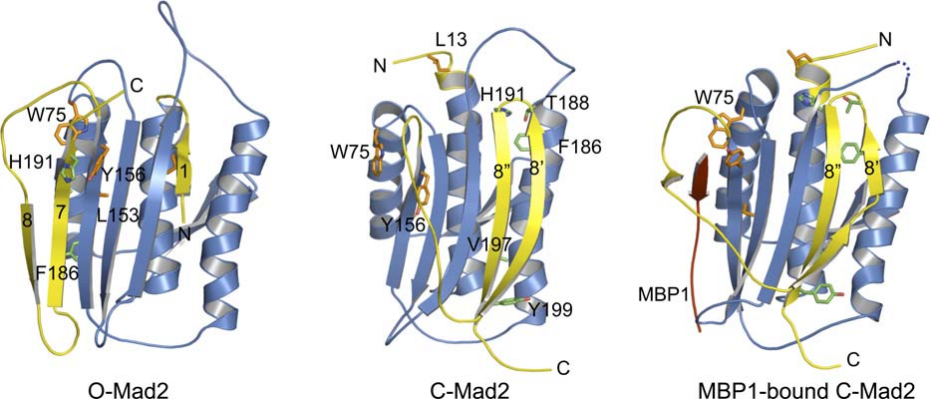
Ribbon Diagram of Various Mad2 Conformers Mad2 and MBP1 are colored blue and red, respectively. The C-terminal region that undergoes a large conformational change from O-Mad2 to C-Mad2 is colored yellow. The N- and C-termini of Mad2 are labeled. The side chains of the conformation-specific Mad2 mutants are shown as sticks. Residues for the O-Mad2-specific mutants (F186A, T188A, H191A, and V197A) are colored green. Residues for the C-Mad2-specific mutants (L13A, W75A, L153A, and Y156A) are colored orange. All structural figures were generated with PyMOL (http://www.pymol.org).

We performed systematic structure-based mutagenesis to identify Mad2 mutants that preferably adopt either the open or closed conformation in the background of the R133A mutation. We used the elution profiles of anion exchange chromatography and NMR spectroscopy to determine the conformational state of the Mad2 mutants and to measure of the O–C conversion rates of mutants that can form both conformers. The binding affinities of these Mad2 mutants toward the Mad2-binding motif of Cdc20 were determined by isothermal titration calorimetry (ITC). The results from these studies are summarized in Table 1. Previous studies showed that a Mad2 mutant with its C-terminal ten residues deleted (Mad2^ΔC^) exclusively adopts the open conformation and can no longer interact with Cdc20 [8,15,17]. The majority of Mad2 mutants formed both O-Mad2 and C-Mad2 conformers that interconverted with rates similar to that of Mad2^R133A^. However, several Mad2 mutants behaved similarly to Mad2^ΔC^ and only adopted the open conformation, including F186A, T188A, H191A, V197A, and Y199A (Figure 2). None of these mutants had detectable binding toward Cdc20 (Table 1). In addition, we identified several Mad2 mutations that selectively destabilized the open conformation of Mad2, such as L13A, W75A, L153A, and Y156A. These mutants preferably adopted the closed conformation (Figure 2). Among these C-Mad2-specific mutants, Mad2^L13A^, Mad2^L153A^, and Mad2^Y156A^ retained their ability to bind to Cdc20 (Table 1), consistent with C-Mad2 being the more active species of Mad2 for Cdc20 binding. Because W75 is located in the ligand-binding site of Mad2, Mad2^W75A^ does not bind to Cdc20 (Figure 2).

**Table 1:**
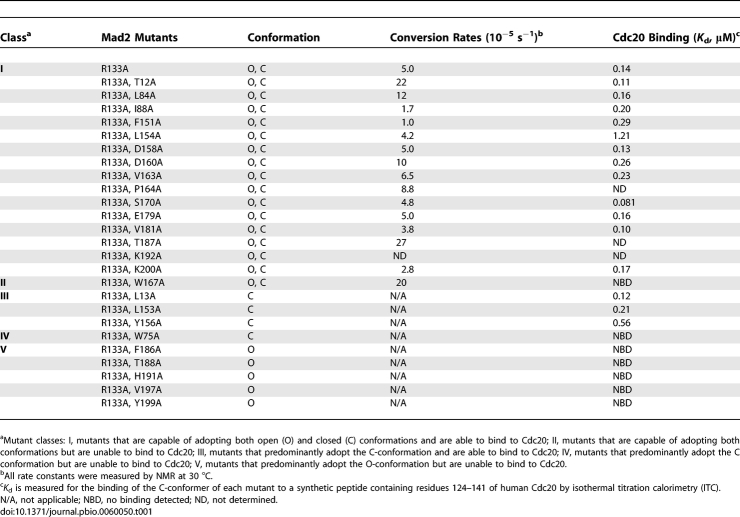
Summary of the Properties of Mad2 Mutants

### Mad2^L13A^ Forms a Symmetric C–C Mad2 Dimer

Because the Mad2^L13A,R133A^ double mutant exclusively adopts the monomeric C-Mad2 conformation, we next introduced the L13A mutation into the wild-type Mad2 (Mad2^WT^) to obtain a symmetric C–C Mad2 dimer. C79 and C106 of Mad2 are located in close proximity and tend to form an intramolecular disulfide bond, causing conformational heterogeneity. To facilitate crystallization, we created a Mad2^L13A,C79S,C106S^ triple mutant, which retained its abilities to bind to Cdc20 and inhibit APC/C in vitro (see below). For simplicity, we will hereafter refer to this triple mutant as Mad2^L13A^. We next fractionated both Mad2^WT^ and Mad2^L13A^ on an anion exchange column (Figure S2A). Similar to Mad2^R133A^, Mad2^WT^ eluted in two well-resolved peaks (Q1 and Q2), which were further fractionated on a gel filtration column. Mad2^WT^ in the low-salt peak (Q1) was monomeric, whereas Mad2 in the high-salt peak (Q2) eluted on the gel filtration column with an apparent molecular mass of about 50 kDa, consistent with it being a dimer (Figure S2B). NMR studies further confirmed that the Mad2^WT^ monomer had the O-Mad2 conformation, and at least one copy of Mad2 in the dimer had the C-Mad2 conformation [17].

In contrast to Mad2^WT^, Mad2^L13A^ eluted as a single high-salt peak on an anion-exchange column (Figure S2A). Mad2^L13A^ in this peak eluted as a dimer from a gel filtration column (Figure S2B). We next used 2D ^1^H-^15^N transverse-relaxation optimized heteronuclear single quantum coherence spectroscopy (TROSY-HSQC) to further characterize the conformational state of Mad2^L13A^. The peaks in the HSQC spectrum of Mad2^L13A^ largely overlap with those in the HSQC spectrum of C-Mad2^R133A^, indicating that Mad2^L13A^ has the C-Mad2 conformation (unpublished data). The HSQC spectrum of the 205-residue Mad2^L13A^ protein has only about 190 backbone peaks, consistent with each backbone amide group of Mad2^L13A^ having a single peak. Thus, the column fractionation profiles and the TROSY-HSQC spectrum of Mad2^L13A^ suggest that Mad2^L13A^ forms a symmetric C–C dimer.

We next used equilibrium sedimentation to determine the native molecular mass of Mad2^L13A^ and to measure its self-association affinity (Figure S2C). After fitting the data to a single ideal species, we obtained a molecular mass of 43.5 kDa, which was about twice the predicted molecular mass of Mad2^L13A^ (23.5 kDa). Fitting the data to a monomer-dimer equilibrium model yielded a dissociation constant (*K*_d_) of 0.25 μM for the Mad2^L13A^ dimer. Thus, Mad2^L13A^ forms a stable symmetric dimer with relatively high affinity.

### Crystal Structure of the Symmetric C–C Mad2^L13A^ Dimer

Our extensive efforts to crystallize the Mad2^WT^ dimer failed, likely due to its conformational heterogeneity. However, we obtained crystals of Mad2^L13A^ that diffracted X-rays to a minimum Bragg spacing of 1.95 Å and determined its structure using molecular replacement. Data collection and refinement statistics are listed in Table 2.

**Table 2:**
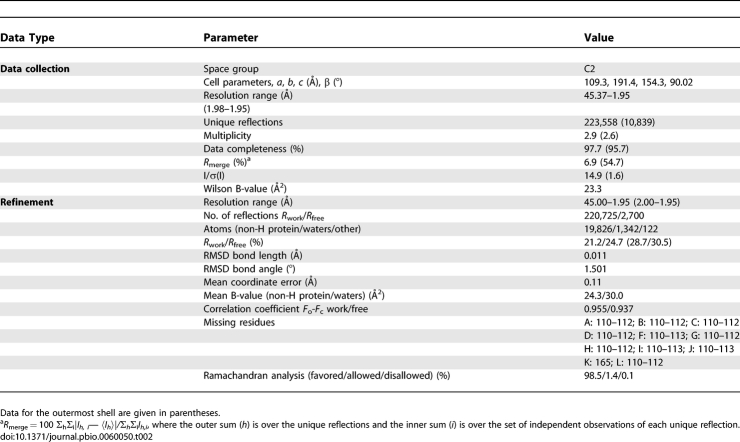
Data Collection and Refinement Statistics

**Figure 3:**
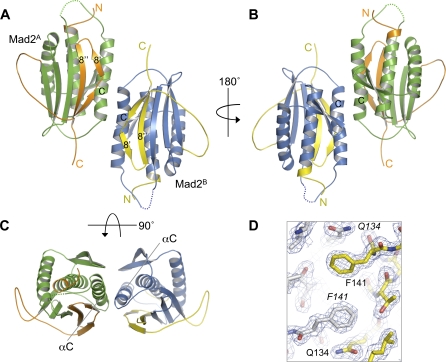
Structural Overview of the Symmetric Mad2^L13A^ Dimer (A–C) Ribbon diagrams of the Mad2^L13A^ dimer in different views. The two Mad2 molecules are named Mad2^A^ and Mad2^B^. The secondary structural elements located at the dimer interface are labeled. Loops not located in the electron density (residues 110–113) are shown as dashed lines. The Mad2 cores are colored green in Mad2^A^ and blue in Mad2^B^. The N- and C-terminal regions involved in the O–C Mad2 conformational change are colored orange in Mad2^A^ and yellow in Mad2^B^. (D) σ_A_-weighted 2*F*_o_-*F*_c_ electron density around residue F141 of Mad2^L13A^ contoured at the 1σ level.

**Figure 4:**
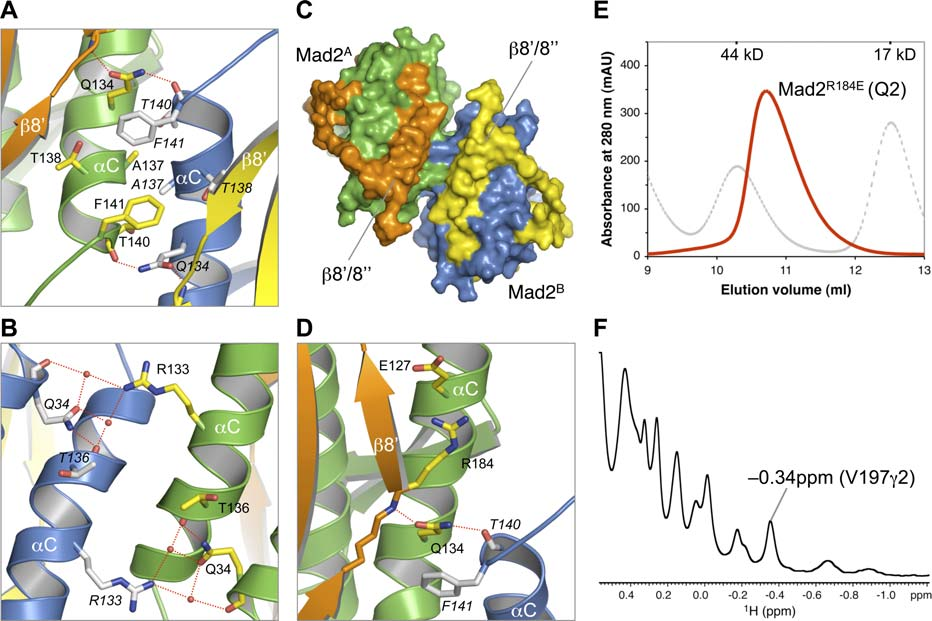
Interactions at the Symmetric Mad2 Dimer Interface (A and B) Interactions between Mad2^A^ and Mad2^B^. The side chains of contacting residues are shown as sticks. Nitrogen and oxygen atoms are colored blue and red, respectively. Mad2^A^ carbons are colored yellow; Mad2^B^ carbons are colored gray and labeled in italics. The tightly bound water molecules are drawn as red spheres. (C) Surface diagram of the Mad2^L13A^ dimer. Same color scheme is used as in Figure 3A–3C. (D) Hydrogen bonds with Q134 in Mad2^A^. Hydrogen bonds are indicated by red dashed lines. (E) Mad2^R184E^ adopts the monomeric C-Mad2 conformation**.** Gel filtration chromatogram of the high-salt peak (275 mM salt, Q2) of Mad2^R184E^ obtained with anion exchange chromatography is shown. Mad2^R184E^ has an apparent molecular mass of about 30 kDa, which is consistent with it being a monomer. The elution profile for Mad2^R184E^ is shown in red, and the elution profile for molecular weight standards is shown in gray. The positions for 44 kDa and 17 kDa markers are indicated. (F) The high-field methyl region of the 1D ^1^H spectrum of Mad2^R184E^. The V197γ2 peak (−0.34 ppm) specific to C-Mad2 is labeled. The line width of the methyl peaks is consistent with C-Mad2^R184E^ being monomeric.

Both monomers in the Mad2^L13A^ dimer adopt the C-Mad2 conformation and are related by noncrystallographic, two-fold symmetry (Figures 3A–3C and S3). The two monomers mainly interact through the C-terminal halves of their αC helices. The high resolution of our structure of Mad2^L13A^ allows clear visualization of side chains as well as several well-ordered water molecules at the dimer interface (Figure 3D). The dimerization interface of Mad2^L13A^ is symmetric and consists of residues from the C-terminal half of αC, R184 from β8′, and Q34 at the C-terminal end of αA (Figure 4). These residues form hydrophobic interactions and extensive networks of water-mediated hydrogen bonds. For example, F141 forms intermolecular interactions with A137, T138, Q134, and F141 (Figure 4A). Bridged by two tightly bound water molecules, R133 from one monomer forms a network of hydrogen bonds with both the backbone and side-chain carbonyl groups of Q34 and the backbone carbonyl of T136 from the neighboring monomer (Figure 4B). The interactions between the two Mad2 monomers observed in our structure are consistent with previous mutagenesis results [23]. Mutations of residues directly located at the dimer interface, including R133, Q134, T140, and F141, have been shown to disrupt Mad2 dimerization.

Residues from β8′ in C-Mad2 do not form intermolecular interactions in the C–C Mad2 dimer (Figure 4C). Residues in β1 in O-Mad2 do not interfere with the interactions at the dimer interface mainly involving the C-terminal end of αC. Why does O-Mad2 not form a symmetric O–O dimer using the same interface as that of the C–C dimer? As discussed above, Q134 is a critical residue at the dimer interface. Its side chain forms an intermolecular hydrogen bond with the backbone carbonyl of T140. The orientation of the Q134 side chain is determined by its packing with F141 from the neighboring monomer and, more importantly, by an intramolecular hydrogen bond with the backbone amide of R184 (Figure 4D). In C-Mad2, R184 is located in a β bulge and forms an electrostatic interaction with E127 on αC, thus presenting its backbone amide for hydrogen bonding with the side chain of Q134. In O-Mad2, R184 is located at the opposite side of the molecule. The side chain of Q134 packs against W100 and is not available for intermolecular hydrogen bonding. Thus, R184 of β8′ indirectly contributes to Mad2 dimerization by forming an intramolecular hydrogen bond with the side chain of Q134, explaining the inability of O-Mad2 to form symmetric dimers. V197 in O-Mad2 is located in the flexible C-terminal tail, whereas it resides in β8′′ and packs against W100 in C-Mad2 [17]. As a consequence, the γ2 methyl group of V197 (V197γ2) has a high-field ^1^H chemical shift at −0.34 parts per million (ppm) only in C-Mad2. Hence the −0.34 ppm V197γ2 peak is unique to C-Mad2. Consistent with the essential role of R184 in symmetric C–C Mad2 dimerization, Mad2^R184E^ (a point mutant of Mad2 with R184 mutated to glutamate in wild-type Mad2) adopts the monomeric C-Mad2 conformation as evidenced by its apparent molecular weight from the gel filtration chromatography and the existence of the unique V197γ2 peak at −0.34 ppm in the 1D NMR spectrum (Figure 4E and 4F).

### Mad2^WT^ Forms Both Symmetric C–C and Asymmetric O–C Dimers

**Figure 5:**
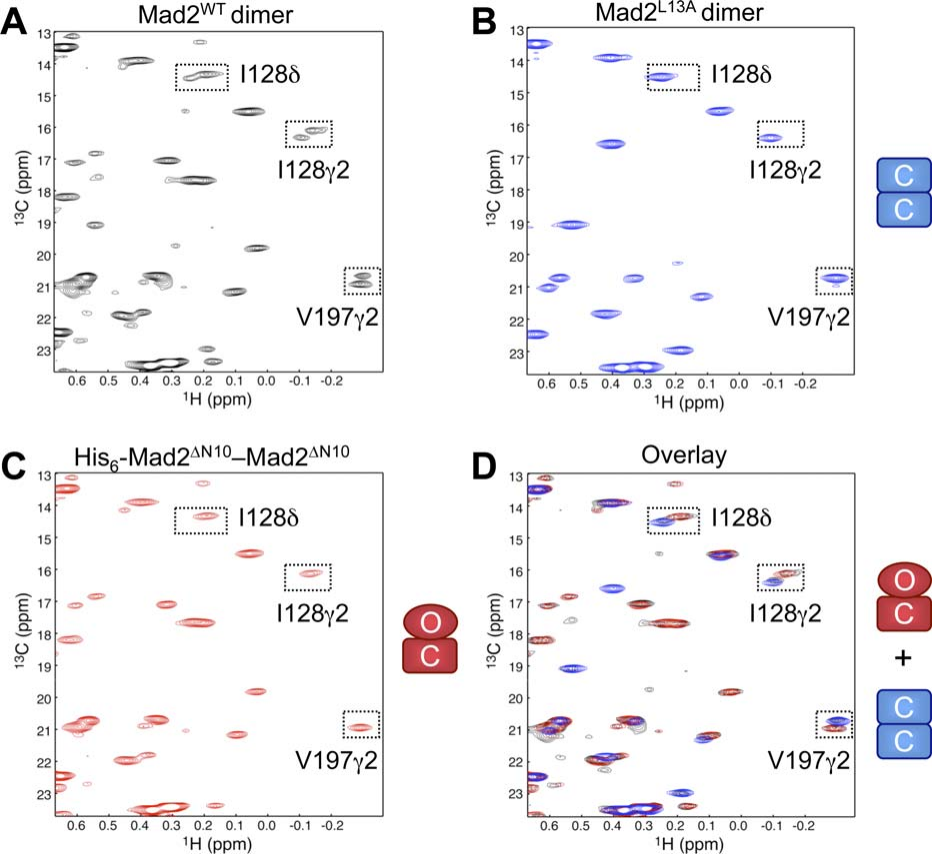
Mad2^WT^ Forms Both Symmetric C–C and Asymmetric O–C Dimers (A) ^1^H-^13^C HSQC spectrum of the Mad2^WT^ dimer with peaks shown in black. The peaks of I128δ, I128γ2, and V197γ2 methyl groups are boxed. (B) ^1^H-^13^C HSQC spectrum of the Mad2^L13A^ C-C dimer with peaks in blue. (C) ^1^H-^13^C HSQC spectrum of the Mad2^ΔN10^ O-C dimer with peaks in red (see Materials and Methods). (D) Overlay of the three ^1^H-^13^C HSQC spectra described in (A–C).

Our previous biochemical and NMR studies have shown that the Mad2^WT^ dimer contains at least one copy of C-Mad2 [17]. However, it is unclear whether the Mad2^WT^ dimer is a symmetric C–C dimer, an asymmetric O–C dimer, or a mixture of both. To characterize the nature of the Mad2^WT^ dimer, we compared its 2D ^1^H-^13^C HSQC spectrum with those of the symmetric C–C Mad2^L13A^ dimer and an asymmetric O–C Mad2 dimer (Figure 5). As discussed above, the −0.34 ppm V197γ2 peak is unique to C-Mad2. The symmetric C–C and asymmetric O–C dimers each contain a single V197γ2 peak at −0.34 ppm. However, the V197γ2 peak in the C–C Mad2 dimer has a higher-field ^13^C chemical shift as compared to that in the O–C Mad2 dimer. The Mad2^WT^ dimer has two peaks for V197γ2, with an intensity ratio of about 1:3 (Figure 5A). The stronger peak overlays well with the V197γ2 peak in the O–C Mad2 dimer, whereas the weaker peak corresponds to the V197γ2 peak in the C–C Mad2 dimer (Figure 5D). Both methyl groups of I128 in the Mad2^WT^ dimer also have two sets of peaks that overlay well with those of the C–C and O–C dimers (Figure 5). Thus, the Mad2^WT^ dimer contains a mixture of symmetric C–C and asymmetric O–C dimers with a molar ratio of about 1:3.

### The Symmetric C–C Mad2 Dimer Is More Active in Inhibiting APC/C^Cdc20^

**Figure 6:**
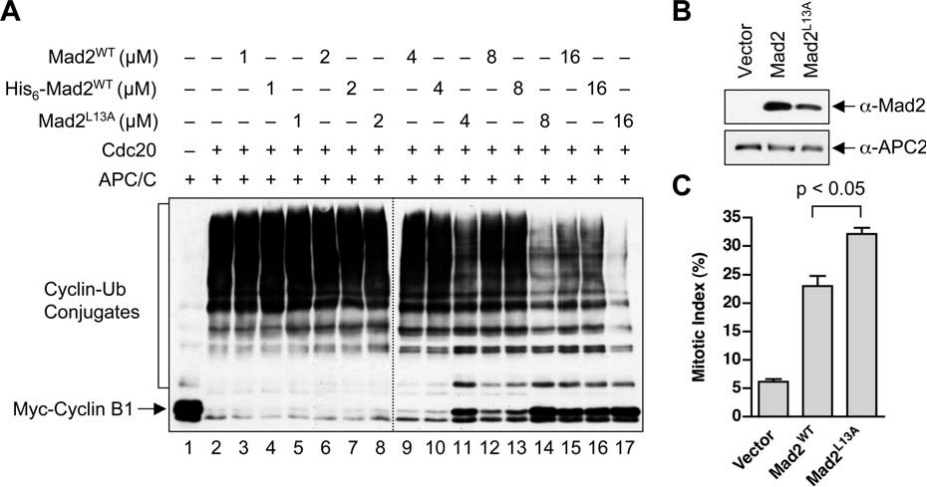
The Mad2^L13A^ Dimer Is More Active Than the Mad2^WT^ Dimer in Inhibiting APC/C^Cdc20^ (A) Mad2^L13A^ is more potent than the Mad2^WT^ dimer in inhibiting APC/C^Cdc20^ in an in vitro reconstituted APC/C ubiquitination assay. Human Cdc20 was incubated with Mad2^WT^ or Mad2^L13^ dimers at varying concentrations (1–16 μM) for 30 min. The mixture was then added to APC/C immunopurified from *Xenopus* egg extracts on anti-APC3 beads for another 1 h. The APC/C beads were then washed and assayed for their ubiquitin ligase activity towards Myc-cyclin B1. The reactions mixtures were blotted with anti-Myc. The unmodified and ubiquitin-conjugated cyclin B1 proteins are indicated. (B) Lysates of HeLa cells transfected with the indicated plasmids were blotted with the indicated antibodies. (C) The mitotic indices of HeLa cells transfected with the indicated plasmids were quantified. At least 400 cells were counted for each transfection. The averages and standard deviations of three separate experiments are shown.

We next compared the APC/C^Cdc20^-inhibitory activities of Mad2^L13A^, untagged dimeric Mad2^WT^, and His_6_-tagged dimeric Mad2^WT^ using an in vitro reconstituted APC/C ubiquitination assay (Figures 6A and S4). Addition of Mad2 to the preformed APC/C^Cdc20^ complex failed to inhibit its activity (unpublished data). Thus, to observe the APC/C^Cdc20^-inhibitory activity of Mad2, we needed to preincubate Mad2 and Cdc20 before the addition of APC/C. When Mad2 and Cdc20 were preincubated for 2 h prior to APC/C addition, Mad2^WT^ and Mad2^L13A^ inhibited APC/C^Cdc20^ with similar potency, with Mad2^L13A^ being slightly more active (Figure S4). Both dimeric untagged and His_6_-tagged Mad2^WT^ behaved similarly in this assay. As a control, Mad2^ΔC^, which lost its ability to bind to Cdc20, had no effect on the activity of APC/C^Cdc20^ (Figure S4). In contrast, when Mad2 and Cdc20 were preincubated for only 30 min prior to their addition to APC/C, Mad2^L13A^ inhibited APC/C^Cdc20^ about 3-fold more potently than did Mad2^WT^ (Figure 6). Therefore, at equilibrium, Mad2^WT^ and Mad2^L13A^ are equally efficient inhibitors of APC/C^Cdc20^. The fact that Mad2^L13A^ inhibits APC/C^Cdc20^ more efficiently than Mad2^WT^ with a shorter preincubation suggests that Mad2^L13A^ has a faster on-rate in Cdc20 binding. Because the majority of dimeric Mad2^WT^ forms the asymmetric O–C dimer, whereas Mad2^L13A^ predominantly forms the symmetric C–C dimer, this finding further suggests that C-Mad2 is more active in APC/C^Cdc20^ inhibition in vitro.

Overexpression of Mad2 causes mitotic arrest in human cells [17]. We next transfected HeLa cells with a control vector or plasmids encoding untagged Mad2^WT^ or Mad2^L13A^. Despite being expressed at slightly lower levels (Figure 6B), Mad2^L13A^ consistently caused a higher percentage of cells to arrest in mitosis than did Mad2^WT^ (Figure 6C). Therefore, as compared to Mad2^WT^, Mad2^L13A^ is more efficient in eliciting mitotic arrest in living cells. Mad2^L13A^ is thus a gain-of-function mutant, suggesting that C-Mad2 is more active than O-Mad2 in APC/C^Cdc20^ inhibition. The ability of Mad2^L13A^ to more effectively titrate p31^comet^ might also contribute to its higher activity in living cells [27].

### The Mad1–Mad2 Core Complex Promotes the Formation of C-Mad2

Vink et al. [28] have recently shown that the in vitro turnover of O-Mad2 bound to purified Mad1–Mad2 core complex has kinetics similar to that of Mad2 turnover at unattached kinetochores in vivo. Thus, the Mad1–Mad2 core complex is the minimal component required for Mad2 turnover and activation at kinetochores. Furthermore, addition of Cdc20 does not appreciably alter the rate of Mad2 turnover on the Mad1–Mad2 core complex, suggesting that Cdc20 binding is not required for the release of Mad2 from the Mad1–Mad2 core complex [28]. However, the conformational state of Mad2 released from the Mad1–Mad2 core complex is unknown.

**Figure 7:**
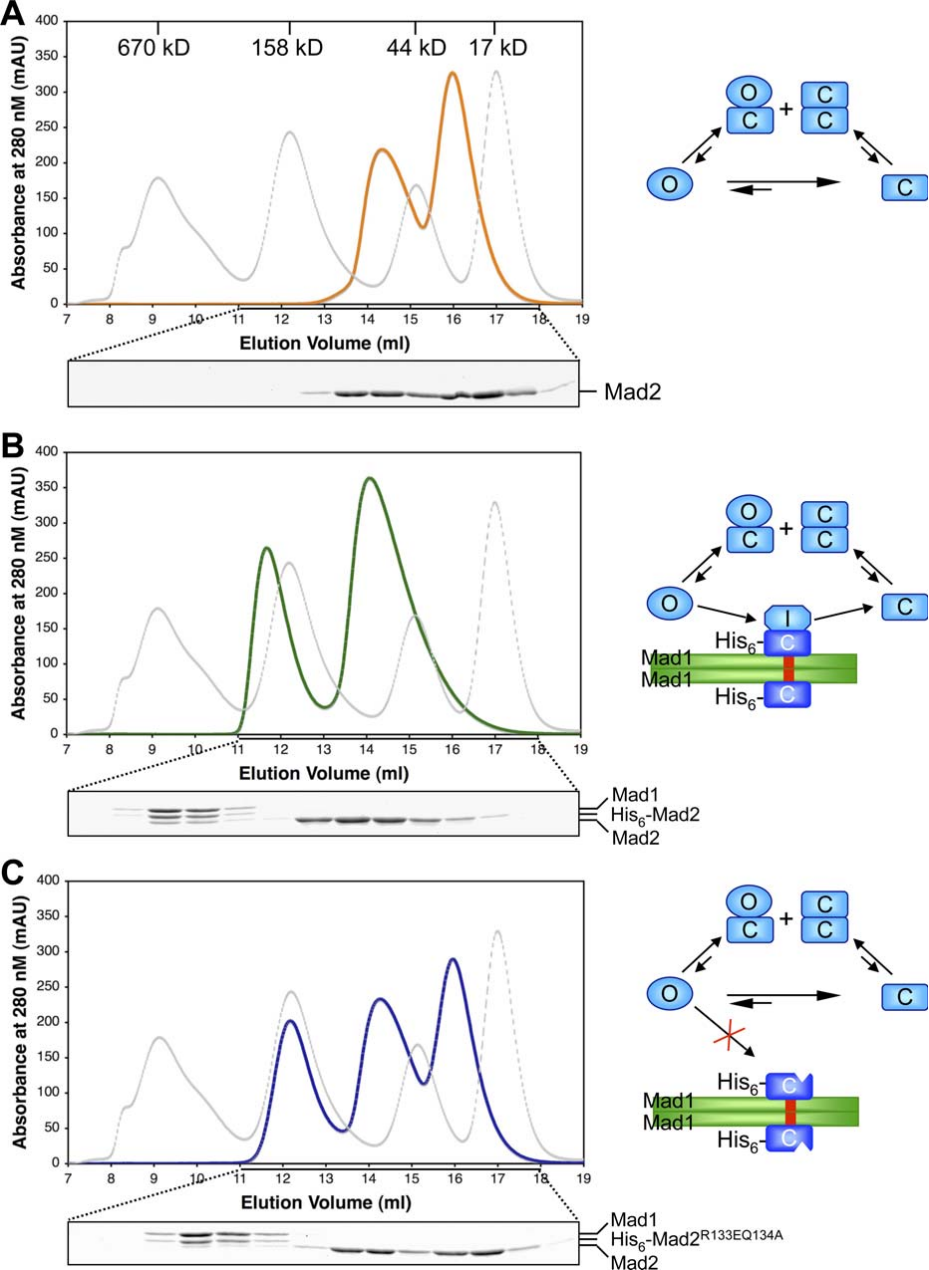
The Mad1–Mad2 Core Complex Promotes the Conversion of O-Mad2 to C-Mad2 (A) The gel filtration chromatogram of O-Mad2 (orange line) incubated with buffer at 37 °C for 30 min. The elution profile of molecular weight standards is shown as a dashed gray line with the native molecular mass of each standard indicated. Coomassie blue–stained SDS-PAGE of fractions with elution volumes from 11 to 18 ml is shown in the bottom panel. The proposed interconversion scheme is shown on the right. The symbols and color scheme are the same as in Figure 1. The same layouts are used in (B) and (C). (B) The gel filtration chromatogram of a protein mixture containing O-Mad2 and the Mad1–Mad2 core complex (green line) incubated at 37 °C for 30 min. (C) The gel filtration chromatogram of a protein mixture containing O-Mad2 and the Mad1–Mad2^R133E,Q134A^ core complex (blue line) incubated at 37 °C for 30 min.

To address this question, we reconstituted Mad2 activation by the Mad1–Mad2 core complex using purified recombinant proteins in solution. We assembled the Mad1–Mad2 core complex by mixing His_6_-Mad2 and the C-terminal fragment of Mad1 (residues 495–718). As a control, we also assembled a Mad1–Mad2 core complex that contained the His_6_-Mad2^R133E,Q134A^ mutant incapable of forming O–C Mad2 dimers. We then incubated untagged ^13^C-labeled O-Mad2 with the Mad1–His_6_-Mad2 or Mad1–His_6_-Mad2^R133E,Q134A^ core complexes at a molar ratio of 4:1 for 30 min at 37 °C. The use of both His_6_-tagged and untagged Mad2 allowed us to distinguish, using SDS-PAGE, the Mad2 molecule in the Mad1–Mad2 core complexes from the free O-Mad2 that turned over on the Mad1–Mad2 core complex. The reaction mixtures were then fractionated by gel filtration chromatography at 4 °C, and the fractions were analyzed using Coomassie blue–stained SDS-PAGE (Figures 7 and S5).

In the absence of the Mad1–Mad2 core complex, about 60% of O-Mad2 remained as monomer while 40% of Mad2 formed dimers (Figure 7A). ^1^H-^13^C HSQC spectra confirmed that the Mad2 monomer adopted the O-Mad2 conformation and that the Mad2 dimer contained a mixture of O–C and C–C Mad2 dimers at a molar ratio of 3:1, as described above (unpublished data). Thus, about 25% of O-Mad2 molecules spontaneously converted to C-Mad2 during the course of the experiment. In the presence of the Mad1–His_6_-Mad2 core complex, about 10% of Mad2 remained bound to the Mad1–Mad2 core complex, while virtually all free Mad2 formed dimers (Figure 7B). Consistent with previous findings, we did not observe substantial dissociation of His_6_-Mad2 from the Mad1–Mad2 core complex. The Mad2 dimer again contained a mixture of O–C and C–C Mad2 dimers at a 3:1 ratio based on ^1^H-^13^C HSQC spectra, indicating that about 60% of O-Mad2 converted to C-Mad2 in the presence of the Mad1–Mad2 core complex. In contrast, addition of the Mad1–His_6_-Mad2^R133E,Q134A^ complex that lost its ability to recruit another copy of O-Mad2 did not appreciably change the rate of conversion from O-Mad2 to C-Mad2 (Figure 7C). Thus, the Mad1–Mad2 core complex promotes the conversion of O-Mad2 to C-Mad2 through O–C Mad2 dimerization. A substantial fraction of Mad2 dissociated from the Mad1–Mad2 core complex adopts the C-Mad2 conformation.

We note that because of the absence of Cdc20 in our assays, unliganded C-Mad2 accumulated to high concentrations and dimerized with a pool of O-Mad2, preventing this pool of O-Mad2 from interacting with the Mad1–Mad2 core complex. In cells, unliganded C-Mad2 is expected to bind to Cdc20 and is unlikely to accumulate to high enough concentrations to compete with the Mad1–Mad2 core complex for O-Mad2. Nevertheless, our results indicate that in the absence of Cdc20, O-Mad2 bound to the Mad1–Mad2 core complex can complete the open-to-closed rearrangement and dissociate from the Mad1–Mad2 core complex as unliganded C-Mad2.

### Release of C-Mad2 from the Mad1–Mad2 Core Complex

**Figure 8:**
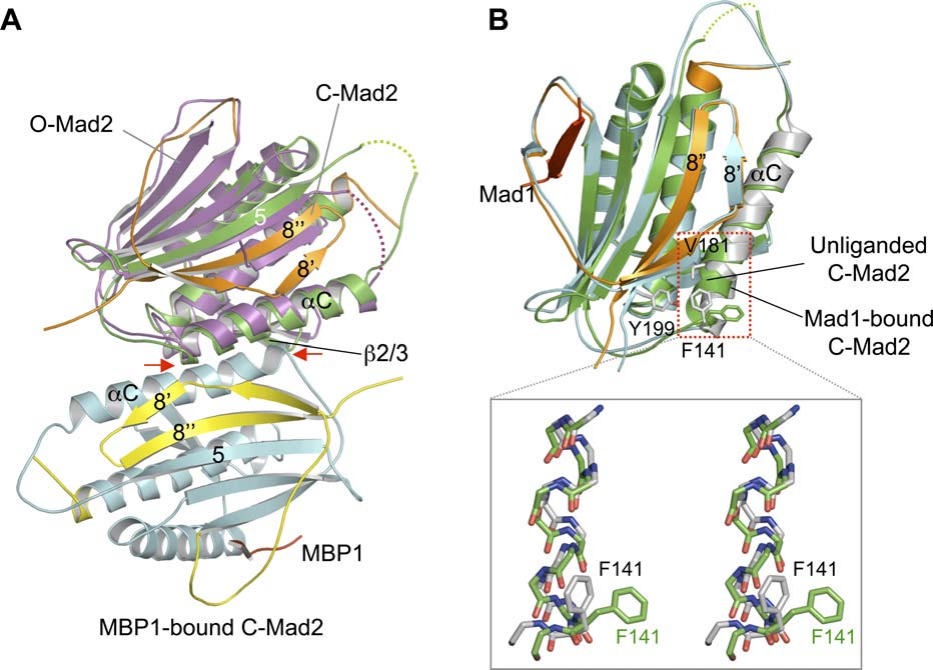
Release of C-Mad2 from the Mad1–Mad2 Core Complex (A) Superposition of C-Mad2 with O-Mad2 in the O–C Mad2 dimer. MBP1-bound C-Mad2 is colored in cyan except for its C-terminal region, which is colored yellow. O-Mad2 in the O–C Mad2 dimer is in magenta. The unliganded C-Mad2 monomer from the Mad2^L13A^ dimer is colored green with its C-terminal region in orange. The steric clashes between C-Mad2 and MBP1-bound Mad2 in this model are indicated by red arrows. (B) Superposition of unliganded C-Mad2 and the Mad1-bound C-Mad2 (top panel). The unliganded C-Mad2 monomer from the Mad2^L13A^ dimer is shown in green. Mad1-Bound C-Mad2 is in cyan with its αC helix colored gray. The side chains of F141, V181, and Y199 in Mad1-bound C-Mad2 are shown as sticks and colored gray while the side chain of F141 of unliganded Mad2 is colored green. The C-terminal ends of the αC helices are boxed with red dashed lines. The bottom panel shows the stereo view of the superposed backbones of residues 135–141 at the C-terminal ends of the αC helices in the two Mad2 molecules. The side chains of F141 in both molecules are shown with the same color schemes as described above.

The Mad1–Mad2 core complex recruits O-Mad2 and converts it to C-Mad2. How is C-Mad2 released from the Mad1–Mad2 core complex after the conversion? Mapelli et al. [25] recently determined the crystal structure of the asymmetric O-Mad2–C-Mad2 dimer. We thus superposed C-Mad2 onto O-Mad2 in the O-Mad2–C-Mad2 dimer (Figure 8A). As described above, a major difference between the fold of O-Mad2 and C-Mad2 is the translocation of the C-terminal region from one side of the molecule to the other, forming the β8′/8′′ hairpin that pairs with β5 in C-Mad2. To accommodate this β hairpin and avoid steric clashes, αC in C-Mad2 needs to rotate slightly, which in turn causes a rotation of the β2/3 hairpin. Consequently, in our structural model, αC of C-Mad2 superposed with O-Mad2 develops steric clashes with β8′ and αA of the original C-Mad2 molecule in the O-Mad2–C-Mad2 dimer (Figure 8A). Thus, C-Mad2 cannot bind to another copy of C-Mad2 using the asymmetric O-Mad2–C-Mad2 dimerization interface. Conversion of O-Mad2 to C-Mad2 on the Mad1–Mad2 core complex introduces steric clashes between αC of the newly formed C-Mad2 and parts of the C-Mad2 molecule in the Mad1–Mad2 core complex, enabling the release of the newly converted C-Mad2.

On the other hand, excluding the ligand-binding site, the structures of unliganded C-Mad2 and Mad1-bound C-Mad2 are highly similar, with a backbone root mean square deviation (RMSD) of 1.1 Å. Furthermore, the ligand-binding site and the symmetric dimerization interface of Mad2 are located on opposite sides of the protein. Why then is C-Mad2 incapable of rebinding to the Mad1–Mad2 core using the symmetric C-Mad2–C-Mad2 interface? A superposition of unliganded C-Mad2 and Mad1-bound C-Mad2 reveals a structural difference in the C-terminal end of their αC helices (Figure 8B). Residues 135–141 in αC adopt an irregular helical conformation in unliganded C-Mad2, whereas they adopt a 3_10_-helical conformation in Mad1-bound C-Mad2. Because of this important difference and a difference in the rotamer conformation of F141, the side chain of F141 points into different directions in the two C-Mad2 structures (Figure 8B). In unliganded C-Mad2, F141 points outward and engages in numerous interactions at the dimerization interface (see Figure 4A). In contrast, F141 in liganded C-Mad2 points inward, forms intramolecular hydrophobic interactions with V181 and Y199, and is unavailable to mediate dimerization (Figure 8B). Mutation of F141 disrupts Mad2 dimerization [23], confirming the essential role of this residue. Thus, ligand binding at one side of Mad2 might trigger structural changes of F141 at the other side, thereby preventing unliganded C-Mad2 from binding to liganded C-Mad2, although we cannot rule out the possibility that the structural differences involving F141 are caused by crystal packing.

### Why Is Unliganded C-Mad2 More Active in APC/C^Cdc20^ Inhibition?

We have shown that unliganded C-Mad2 is more active than O-Mad2 in APC/C^Cdc20^ inhibition in vitro. Because O-Mad2 and unliganded C-Mad2 form the same C-Mad2–Cdc20 complex, the difference in their APC/C^Cdc20^-inhibitory activity is likely caused by different on-rates during their binding to Cdc20. Binding of Cdc20 to O-Mad2 is a complicated process and can be conceptually separated into four steps, not necessarily in the stated order (Figure S6). First, β8 dissociates from β6; the C-terminal region of Mad2 either retains the β7/8 hairpin or possibly rearranges into the β8′/8′′ hairpin as in C-Mad2. Second, β1 dissociates from β5, traverses through the β5-αC loop, and forms an additional turn in αA. Third, the Mad2-binding motif of Cdc20 forms a β strand that pairs with β6 and extends the main β sheet of Mad2. Fourth, the β8′/8′′ hairpin wraps around Cdc20 and translocates to pair with β5, thus trapping Cdc20 in the closed seatbelt conformation. O-Mad2 is thus an autoinhibited conformation in which β8 blocks the accessibility of β6 and, hence, ligand-binding through an intramolecular interaction. Consistent with this notion, a Mad2 deletion mutant (Mad2^1–160^) that lacks β7, β8, and the C-terminal tail still folds, exhibits cooperative unfolding with a melting temperature of 47 °C, and retains weak binding to MBP1 (Figure S7), possibly through the formation of edge-on interactions between β6 and MBP1. In contrast, Mad2^ΔC^ lacks only the C-terminal tail, but retains β7 and β8. This mutant fails to bind to MBP1 because of the blockage of β6 by β8 (unpublished data).

We propose two nonexclusive models to explain why unliganded C-Mad2 is more active in APC/C^Cdc20^ inhibition than O-Mad2 (Figure S6). In the first model (pathway **a**), dissociation of β1 and its subsequent traversing through the β5-αC loop are rate-limiting steps in the conversion of O-Mad2 to I-Mad2. These structural changes involving β1 have already occurred in C-Mad2. The energetic barrier between C-Mad2 and I-Mad2 may be lower than that between O-Mad2 and I-Mad2 (Figure 1). Thus, C-Mad2 can reach the I-Mad2 conformation more easily than O-Mad2, explaining why C-Mad2 is more active in APC/C^Cdc20^ inhibition. In the second model (pathway **b**), because β6 is exposed in C-Mad2, but blocked in O-Mad2, the Mad2-binding motif of Cdc20 more readily forms an edge-on interaction with β6 of C-Mad2. Binding of Cdc20 on one side of Mad2 allosterically triggers the dissociation of the β8′/8′′ hairpin from β5 on the other side of Mad2. This hairpin then wraps around Cdc20 and completes the binding event.

Although only C-Mad2 can form symmetric dimers, the β8′/8′′ hairpin of C-Mad2 does not directly participate in this symmetric dimerization. Formation of symmetric C-Mad2–C-Mad2 dimers does not impede the dissociation of β8′/8′′ from β5 and the binding of C-Mad2 to Cdc20. In contrast, the β8′/8′′ hairpin of C-Mad2 is a major structural element that mediates the binding of O-Mad2. Formation of the asymmetric O-Mad2–C-Mad2 impedes the dissociation of β8′/8′′ from β5 and, hence, the binding of C-Mad2 to Cdc20, explaining the dominant-negative effects of O-Mad2 on C-Mad2. Furthermore, O-Mad2 in the O-Mad2–C-Mad2 dimer is less active in APC/C^Cdc20^ inhibition than C-Mad2, suggesting that O-Mad2 cannot be activated by unliganded C-Mad2 to become I-Mad2, unlike O-Mad2 bound to the Mad1–Mad2 core complex.

### Conclusion

The two-state behavior of Mad2 was discovered nearly a decade ago [8]. It was shown that dimeric Mad2 was active in APC/C^Cdc20^ inhibition. Monomeric Mad2 not only was inactive in APC/C^Cdc20^ inhibition, but also blocked the ability of dimeric Mad2 to inhibit APC/C^Cdc20^ in a dominant-negative manner. We have now determined the crystal structure of an active dimeric Mad2 species, and show that the active Mad2 dimer is a symmetric C-Mad2–C-Mad2 dimer. O-Mad2 forms an asymmetric O-Mad2–C-Mad2 dimer and blocks the ability of C-Mad2 to inhibit APC/C^Cdc20^ in a dominant-negative manner. The Mad1–Mad2 core complex catalyzes the conversion of O-Mad2 to unliganded C-Mad2 in the absence of Cdc20.

Our results further support the following conformational activation model for Mad2-dependent spindle checkpoint signaling (Figure 1). In this model, cytosolic O-Mad2 is autoinhibited and has a high kinetic barrier for binding to Cdc20. Upon checkpoint activation, O-Mad2 is recruited to kinetochore-bound Mad1–Mad2 core complex through asymmetric O-Mad2–C-Mad2 dimerization. The Mad1–Mad2 core complex converts O-Mad2 to a short-lived intermediate Mad2 (I-Mad2). I-Mad2 is kinetically more favorable for Cdc20 binding and can bind directly to Cdc20 to form C-Mad2. Alternatively, I-Mad2 can convert to unliganded C-Mad2 on its own and, upon release from the Mad1–Mad2 core complex, can form symmetric C-Mad2–C-Mad2 dimers. Both monomeric C-Mad2 and symmetric C-Mad2–C-Mad2 dimer are active in APC/C^Cdc20^ inhibition.

## Materials and Methods

### Protein expression and purification.

The coding region of human Mad2 was amplified by polymerase chain reaction (PCR) and cloned into either a pGEX-KT or pQE30 (Qiagen) vector, each of which also included a tobacco etch virus (TEV) protease cleavage site. Mad2 mutants were generated with the QuikChange mutagenesis kit (Stratagene). The pQE30-Mad2 plasmids were transformed into the bacteria strain M15[pREP4] to produce various His_6_-tagged Mad2 proteins. These proteins were purified with Ni^2+^-NTA agarose resin (Qiagen) and cleaved with TEV protease to remove the His_6_-tag. The proteins were further purified by anion exchange chromatography followed by gel filtration chromatography. Expression of pGEX-Mad2^L13A,C79S,C106S^ (referred to as Mad2^L13A^ for simplicity) in the bacterial strain BL21 produced a GST-Mad2 fusion protein. The fusion protein was isolated with glutathione-Sepharose beads (GE Healthcare) and cleaved with TEV protease to remove GST. The Mad2^L13A^ protein was further purified by anion exchange and gel filtration chromatography. The purified Mad2^L13A^ dimer was concentrated to 3 mg/ml in a buffer containing 20 mM Tris (pH 8.0), 50 mM NaCl, and 2 mM TCEP.

To prepare the asymmetric Mad2^ΔN10^ O–C dimer, we first expressed and purified the His_6_-Mad2^ΔN10^ monomer in the O-Mad2 conformation. We had previously shown that O-Mad2 was stable at 4 °C, whereas it underwent slow spontaneous conversion to C-Mad2 at 30 °C [17]. Incubation of O-Mad2^ΔN10^ with TEV overnight at 4 °C did not result in the cleavage of the His_6_-tag from His_6_-O-Mad2^ΔN10^, whereas TEV efficiently cleaved other unrelated His_6_-tag proteins under the same conditions. This result suggested that the TEV cleavage site in His_6_-O-Mad2^ΔN10^ was not accessible. We thus incubated the mixture of His_6_-O-Mad2^ΔN10^ and TEV overnight at 30 °C, which resulted in the cleavage of about 50% of the His_6_-O-Mad2^ΔN10^ molecules. This mixture of His_6_-tagged and untagged Mad2^ΔN10^ fractionated as a single high-salt peak on an anion exchange column and as a 1:1 heterodimer on a gel filtration column. Moreover, the ^1^H-^13^C HSQC spectrum of the Mad2^ΔN10^ dimer was virtually identical to that of O-Mad2^ΔC^–C-Mad2^WT^ dimer, indicating that Mad2^ΔN10^ indeed formed an O-Mad2–C-Mad2 dimer. We reasoned that as His_6_-O-Mad2^ΔN10^ spontaneously converted to His_6_-C-Mad2^ΔN10^ or an intermediate Mad2 state, its TEV cleavage site became accessible, resulting in the cleavage of the His_6_-tag in this population of Mad2^ΔN10^. The formation of the His_6_-O-Mad2^ΔN10^–untagged C-Mad2^ΔN10^ dimer prevented the further conversion of His_6_-O-Mad2^ΔN10^ to His_6_-C-Mad2^ΔN10^, and thus prevented further cleavage of the His_6_-tag in the rest of the Mad2^ΔN10^ molecules by TEV.

### Crystallization, data collection, and structure determination.

The Mad2^ L13A^ dimer was crystallized at 20 °C using the sitting-drop vapor-diffusion method. Drops were formed by mixing 1 μl of protein and 1 μl of reservoir solution that contained 19% (w/v) PEG 2000, 16% (v/v) glycerol, 100 mM Tris (pH 8.0), and 0.3 M MgCl_2_. Larger crystals were obtained by seeding using the same conditions. The crystals were cryoprotected with reservoir solution and then flash-cooled in liquid propane. Crystals diffracted to a minimum Bragg spacing (*d*_min_) of about 1.9 Å. At lower resolution, the diffraction data are compatible with an orthorhombic crystal symmetry. However, at higher resolution, the crystals exhibited the symmetry of space group C2 with cell dimensions of *a* = 109 Å, *b* = 191 Å, *c* = 154 Å and β = 90.02° with 12 molecules per asymmetric unit.

Diffraction data were collected at beamline 19-ID (SBC-CAT) at the Advanced Photon Source (Argonne National Laboratory, Argonne, Illinois, United States) and processed with HKL2000 [29]. The Mad2^L13A^ dimer structure was determined by the molecular replacement method with the program Phaser [30] using the Mad2 core (residues 12–36, 58–158, and 177–205) from the structure of Mad2–MBP1 as the search model. Refinement was performed with REFMAC5 [31] from the CCP4 package [32] using diffraction data to a resolution of 1.95 Å, interspersed with manual rebuilding using the program Coot [33]. The 12 molecules in the asymmetric unit are arranged in two sets of six molecules related by almost perfect translational symmetry. No noncrystallographic symmetry restraints were used during refinement. Between one and four residues per Mad2 molecule were disordered and were not included in the model. The final model (*R*_work_ = 21.2% and *R*_free_ = 24.7%) contains 2,464 residues, 1,342 water molecules, eight magnesium ions, 32 chloride ions, as well as ten short PEG molecules. All but two residues are in the favored region of the Ramachandran plot. The two residues in the disallowed region are located at surface loops and are associated with weak electron density. Data collection and structure refinement statistics are summarized in Table 2.

### Analytical ultracentrifugation and isothermal titration calorimetry.

Sedimentation equilibrium experiments were performed at 4 °C with a Beckman Optima XL-I analytical ultracentrifuge using a four-position An60Ti rotor with six-channel equilibrium centerpieces (optical path length = 1.2 cm) and an absorbance optical detection system (Beckman Instruments). Sample channels were filled with 100 μl of protein at three different concentrations (0.23, 0.36, and 0.50 mg/ml) in a buffer containing 20 mM Tris (pH 8.0), 50 mM NaCl, 0.2 mM TCEP. The reference channels were filled with 110 μl of buffer. The absorbance at 280 nm was monitored for each cell in 0.002-cm steps. Samples were centrifuged at 13,000 rpm, 17,500 rpm, and 25,000 rpm until equilibrium had been reached, followed by overspeed runs at 42,000 rpm to obtain baseline values of absorbance. The partial specific volume (0.7451 ml/g) and the solvent density (1.0054 g/ml) were calculated using the program SEDNTERP (http://rd.plos.org/pbio.0060050). Sedimentation equilibrium datasets were fitted to the self-association model using Beckman Optima XL-A/Xl-I data analysis software (Origin 6.03). A global analysis was carried out for datasets obtained at different concentrations and rotor speeds. Isothermal titration calorimetry was performed as described [27].

### NMR spectroscopy.

ll NMR spectra were acquired at 30 °C on a Varian Unity Inova 800 MHz spectrometer using H_2_O/D_2_O 95:5 (v/v) as the solvent. Samples typically contained 0.1 mM protein in a buffer consisting of 50 mM phosphate (pH 6.8), 300 mM KCl, and 1 mM DTT.

### Mammalian tissue culture, transfection, and APC/C assays.

HeLa Tet-on (Invitrogen) cells were cultured in DMEM medium supplemented with 10% fetal bovine serum. The cells were transfected with pCS2-Mad2 vectors using Effectene (Qiagen). After 36 h, the cells were stained with Hoechst 33342 (Molecular Probes) and examined using an inverted fluorescence microscope (Zeiss). Lysates of the transfected cells were blotted using the appropriate antibodies. APC/C assays were performed as described [34,35].

## Supporting Information

Figure S1 Electrostatic Potential Surfaces of O-Mad2 and C-Mad2 Surface representations for O-Mad2 and C-Mad2 in similar orientations. Positive and negative electrostatic potentials are colored blue and red, respectively. The negatively charged patch around β6 in C-Mad2 is circled.
(2.6 MB TIF)

Figure S2 Dimerization Properties of Mad2^WT^ and Mad2^L13A^
(A) Anion exchange chromatograms of Mad2^WT^ (top panel) and Mad2^L13A^ (bottom panel). The salt concentrations in which each sample eluted are indicated. The Coomassie blue-stained SDS-PAGE of column fractions are shown below the corresponding chromatogram.
(B) Gel filtration chromatograms of Mad2^WT^ in the Q1 peak (top panel) as described in (A), Mad2^WT^ in the Q2 peak (middle panel), and Mad2^L13A^ (bottom panel). The elution profile of molecular weight standards is shown as a dashed gray line with the native molecular mass of each standard indicated.
(C) Equilibrium sedimentation analysis of Mad2^L13A^. Datasets were collected at centrifugation speeds of 13,000 rpm (black), 17,500 rpm (red), and 25,000 rpm (green). The plots of the best fits (bottom panel) and their residuals (top panel) were generated by fitting the data to a monomer-dimer equilibrium model. The triangles and squares denote samples at 0.36 mg/ml and 0.50 mg/ml concentrations, respectively.
(5 MB TIF)

Figure S3 Two Types of Interfaces between Mad2^L13A^ Protomers The asymmetric unit of the Mad2^L13A^ crystals contains 12 monomers. The pairwise backbone root mean square deviations (RMSD) for the 12 monomers are below 0.5 Å. A group of four Mad2 protomers (named Mad2^A^, Mad2^B^, Mad2^C^, and Mad2^D^) is shown in ribbon representations to display the two types of molecular interfaces observed in the Mad2^L13A^ structure. The color code is as follows: Mad2^A^ is green, Mad2^B^ is blue, Mad2^C^ is magenta, and Mad2^D^ is orange. Mad2^A^ and Mad2^B^, as well as Mad2^C^ and Mad2^D^, are related by a noncrystallographic two-fold axis. In one interface, Mad2^A^ and Mad2^B^ interact with each other mainly through the C-terminal halves of the αC helices. In the other interface, the N-terminal regions of Mad2^C^ and Mad2^D^ insert into the ligand-binding pockets of Mad2^A^ and Mad2^B^, respectively. This tetrameric arrangement likely does not reflect the oligomeric status of Mad2^L13A^, as it exists predominantly as a dimer in solution, based on gel filtration and equilibrium sedimentation experiments (Figure S2). Furthermore, mutations of several residues on αC, including R133A, completely disrupt Mad2 dimerization in solution, indicating that αC is the major structural determinant for Mad2 dimerization. Finally, the N-terminal region of Mad2 does not share sequence homology with the Mad2-binding consensus motifs and is dispensable for dimer formation. Therefore, the interactions between the N-terminal regions of Mad2^C,D^ and the ligand-binding sites of Mad2^A,B^ are very likely a result of crystal packing. Though these types of interactions are unlikely to be functionally relevant, they may explain the ability of Mad2 dimers to form higher-order oligomers at high concentrations.
(3.8 MB TIF)

Figure S4 Both Mad2^WT^ and Mad2^L13A^ Dimers Inhibit APC/C Efficiently with Longer Preincubation of Cdc20 and Mad2 Human Cdc20 was incubated with Mad2^WT^ or Mad2^L13A^ dimers at varying concentrations (1–16 μM) for 2 h. The mixture was then added to APC/C immunopurified from *Xenopus* egg extracts on anti-APC3 beads for another 1 h. The APC/C beads were then washed and assayed for their ubiquitin ligase activity towards Myc-cyclin B1. The reactions mixtures were blotted with anti-Myc. The unmodified and ubiquitin-conjugated cyclin B1 proteins are indicated.
(1.4 MB TIF)

Figure S5 The Mad1–Mad2 Core Complex Promotes the Conversion of O-Mad2 to C-Mad2 Overlay of the gel filtration chromatograms shown in Figure 7. “Core + O-Mad2” (green): the protein mixture containing O-Mad2 and the Mad1–Mad2 core complex incubated at 37 °C for 30 min; “Core control + O-Mad2” (blue): the protein mixture containing O-Mad2 and the Mad1–Mad2^R133E,Q134A^ core complex incubated at 37 °C for 30 min; “Buffer + O-Mad2” (orange): O-Mad2 incubated with buffer at 37 °C for 30 min; “Standards” (gray): molecular weight standards are shown with a dashed gray line with the native molecular mass of each standard indicated; and “Core” (red): the Mad1–Mad2 core complex alone.
(3 MB TIF)

Figure S6 Proposed Mechanisms for the Binding of Cdc20 to O-Mad2 or Unliganded C-Mad2 Topology diagrams that illustrate the structural changes in different Mad2 species and the proposed pathways for their binding to Cdc20 are shown. The secondary structural elements are labeled. The core domain for Mad2 is colored blue. The N- and C-terminal regions involved in the Mad2 conformational change are colored yellow, except for residues 172–175 and 184–192, which are shown in green. Cdc20 is shown in red.
(694 kB TIF)

Figure S7 Mad2^1–160^ Undergoes Cooperative Unfolding and Retains Binding to MBP1 (A) The temperature-induced denaturation curves of Mad2^ΔN10^ (shown as solid triangles) and Mad2^1–160^ (solid squares) as monitored by the intensity of the circular dichroism (CD) signal at 220 nm.
(B) Overlay of ^1^H-^15^N HSQC spectra of free Mad2^1–160^ (black) and Mad2^1–160^ in complex with MBP1 (red). The peaks in the Mad2^1–160^ spectra are well dispersed, indicating that Mad2^1–160^ is folded. Several peaks undergo significant chemical shift changes upon the addition of MBP1, indicating that Mad2^1–160^ binds to MBP1.
(1.9 MB TIF)

### Accession Numbers

The atomic coordinates and structure factors for the symmetric C–C Mad2^L13A^ dimer have been deposited in the Protein Data Bank (http://www.rcsb.org/pdb/home/home.do) with accession number PDB ID 2VFX. The Protein Data Bank accession numbers for other proteins discussed in this paper are as follows: Mad1-bound C-Mad2 (PDB ID 1GO4), Mad2–MBP1 (PDB ID 1KLQ), and O–C Mad2 dimer (PDB ID 2V64).

## Abbreviations

APC/C: anaphase-promoting complex or cyclosome
C–C: closed Mad2–closed Mad2
C-Mad2: closed Mad2
HSQC: heteronuclear single quantum coherence
I-Mad2: intermediate Mad2
MBP1: Mad2-binding peptide 1
NMR: nuclear magnetic resonance
O–C: open Mad2–closed Mad2
O-Mad2: open Mad2
ppm: parts per million
