# Supplementary figures and images for: Insights into Mad2 Regulation in the Spindle Checkpoint Revealed by the Crystal Structure of the Symmetric Mad2 Dimer
Source: PLoS Biol. 2008 Mar 4;6(3):e50. doi: 10.1371/journal.pbio.0060050 (PMC2270309; doi:10.1371/journal.pbio.0060050)

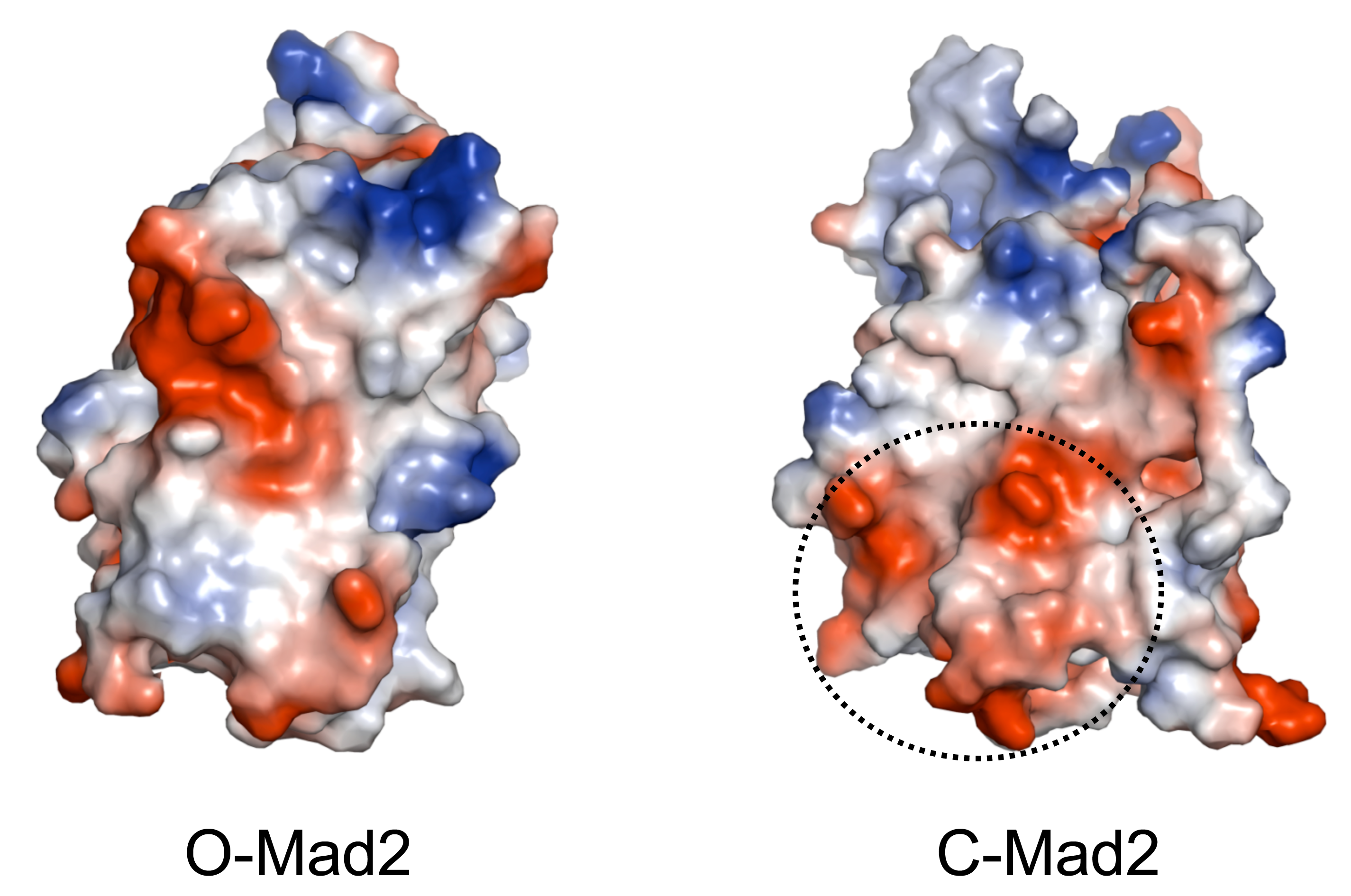

Supplement: Figure S1 — Surface representations for O-Mad2 and C-Mad2 in similar orientations. Positive and negative electrostatic potentials are colored blue and red, respectively. The negatively charged patch around β6 in C-Mad2 is circled. (2.6 MB TIF) [file pbio.0060050.sg001.tif]

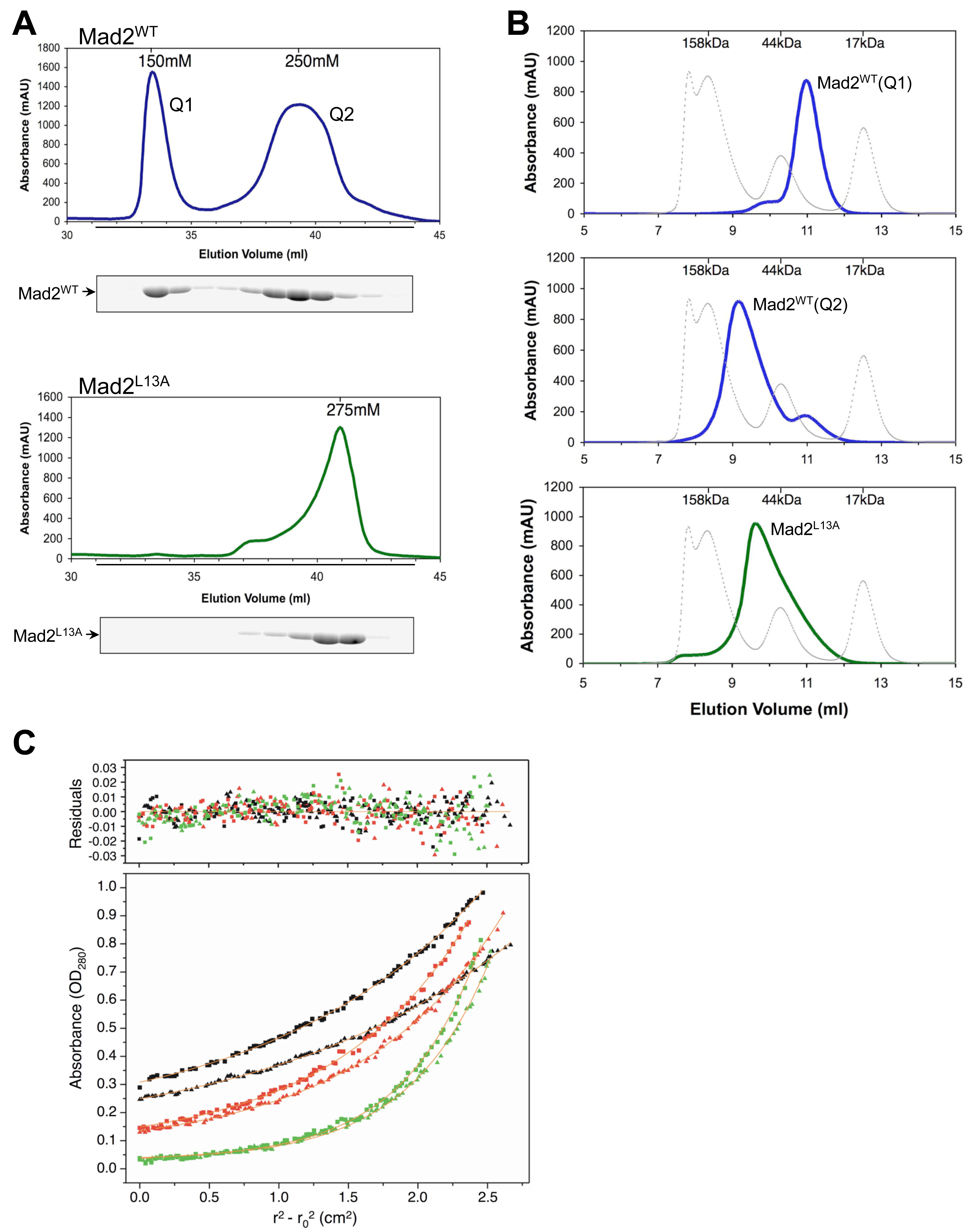

Supplement: Figure S2 — (A) Anion exchange chromatograms of Mad2WT (top panel) and Mad2L13A (bottom panel). The salt concentrations in which each sample eluted are indicated. The Coomassie blue-stained SDS-PAGE of column fractions are shown below the corresponding chromatogram. (B) Gel filtration chromatograms of Mad2WT in the Q1 peak (top panel) as described in (A), Mad2WT in the Q2 peak (middle panel), and Mad2L13A (bottom panel). The elution profile of molecular weight standards is shown as a dashed gray line with the native molecular mass of each standard indicated. (C) Equilibrium sedimentation analysis of Mad2L13A. Datasets were collected at centrifugation speeds of 13,000 rpm (black), 17,500 rpm (red), and 25,000 rpm (green). The plots of the best fits (bottom panel) and their residuals (top panel) were generated by fitting the data to a monomer-dimer equilibrium model. The triangles and squares denote samples at 0.36 mg/ml and 0.50 mg/ml concentrations, respectively. (5 MB TIF) [file pbio.0060050.sg002.tif]

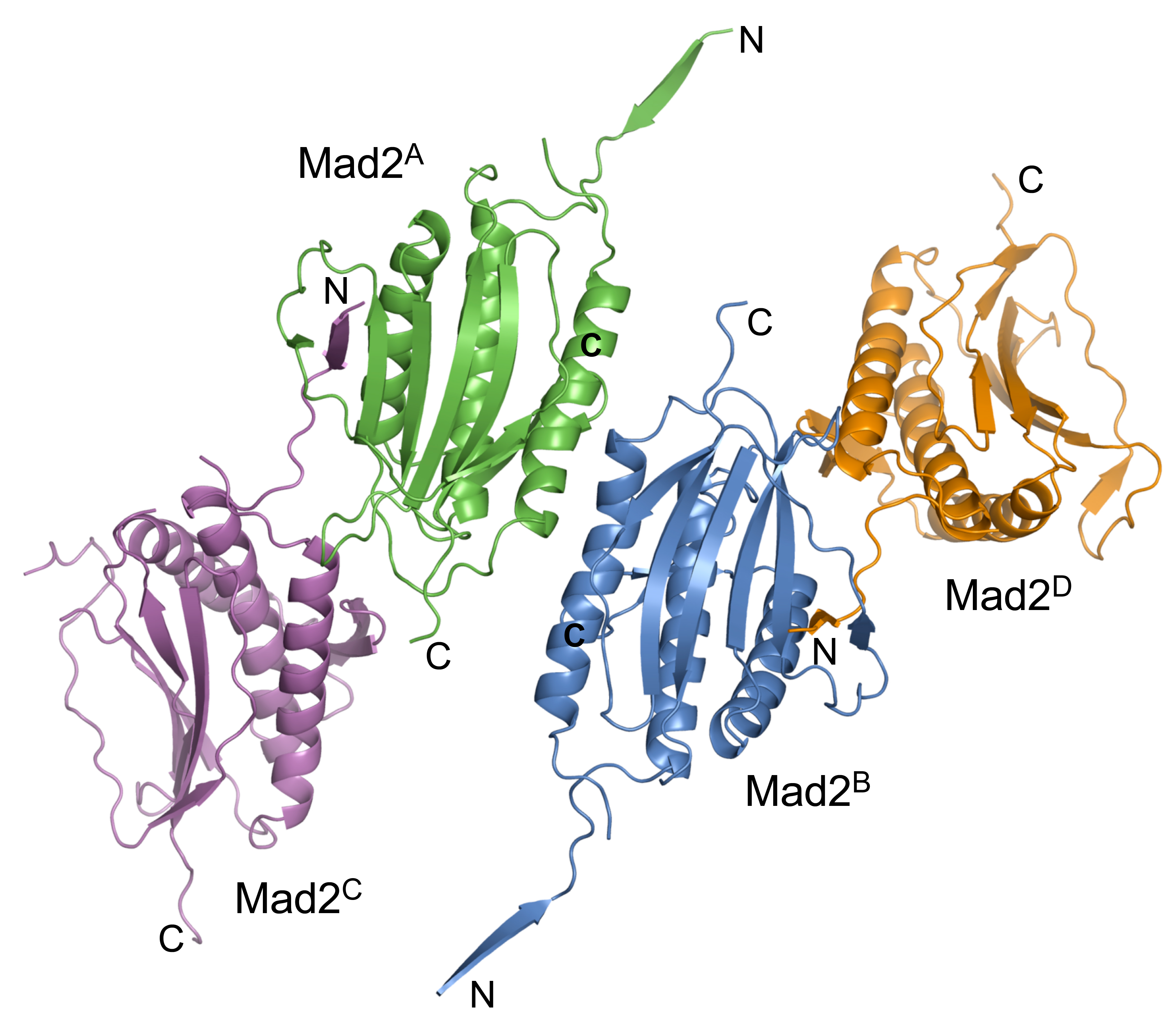

Supplement: Figure S3 — The asymmetric unit of the Mad2L13A crystals contains 12 monomers. The pairwise backbone root mean square deviations (RMSD) for the 12 monomers are below 0.5 Å. A group of four Mad2 protomers (named Mad2A, Mad2B, Mad2C, and Mad2D) is shown in ribbon representations to display the two types of molecular interfaces observed in the Mad2L13A structure. The color code is as follows: Mad2A is green, Mad2B is blue, Mad2C is magenta, and Mad2D is orange. Mad2A and Mad2B, as well as Mad2C and Mad2D, are related by a noncrystallographic two-fold axis. In one interface, Mad2A and Mad2B interact with each other mainly through the C-terminal halves of the αC helices. In the other interface, the N-terminal regions of Mad2C and Mad2D insert into the ligand-binding pockets of Mad2A and Mad2B, respectively. This tetrameric arrangement likely does not reflect the oligomeric status of Mad2L13A, as it exists predominantly as a dimer in solution, based on gel filtration and equilibrium sedimentation experiments (Figure S2). Furthermore, mutations of several residues on αC, including R133A, completely disrupt Mad2 dimerization in solution, indicating that αC is the major structural determinant for Mad2 dimerization. Finally, the N-terminal region of Mad2 does not share sequence homology with the Mad2-binding consensus motifs and is dispensable for dimer formation. Therefore, the interactions between the N-terminal regions of Mad2C,D and the ligand-binding sites of Mad2A,B are very likely a result of crystal packing. Though these types of interactions are unlikely to be functionally relevant, they may explain the ability of Mad2 dimers to form higher-order oligomers at high concentrations. (3.8 MB TIF) [file pbio.0060050.sg003.tif]

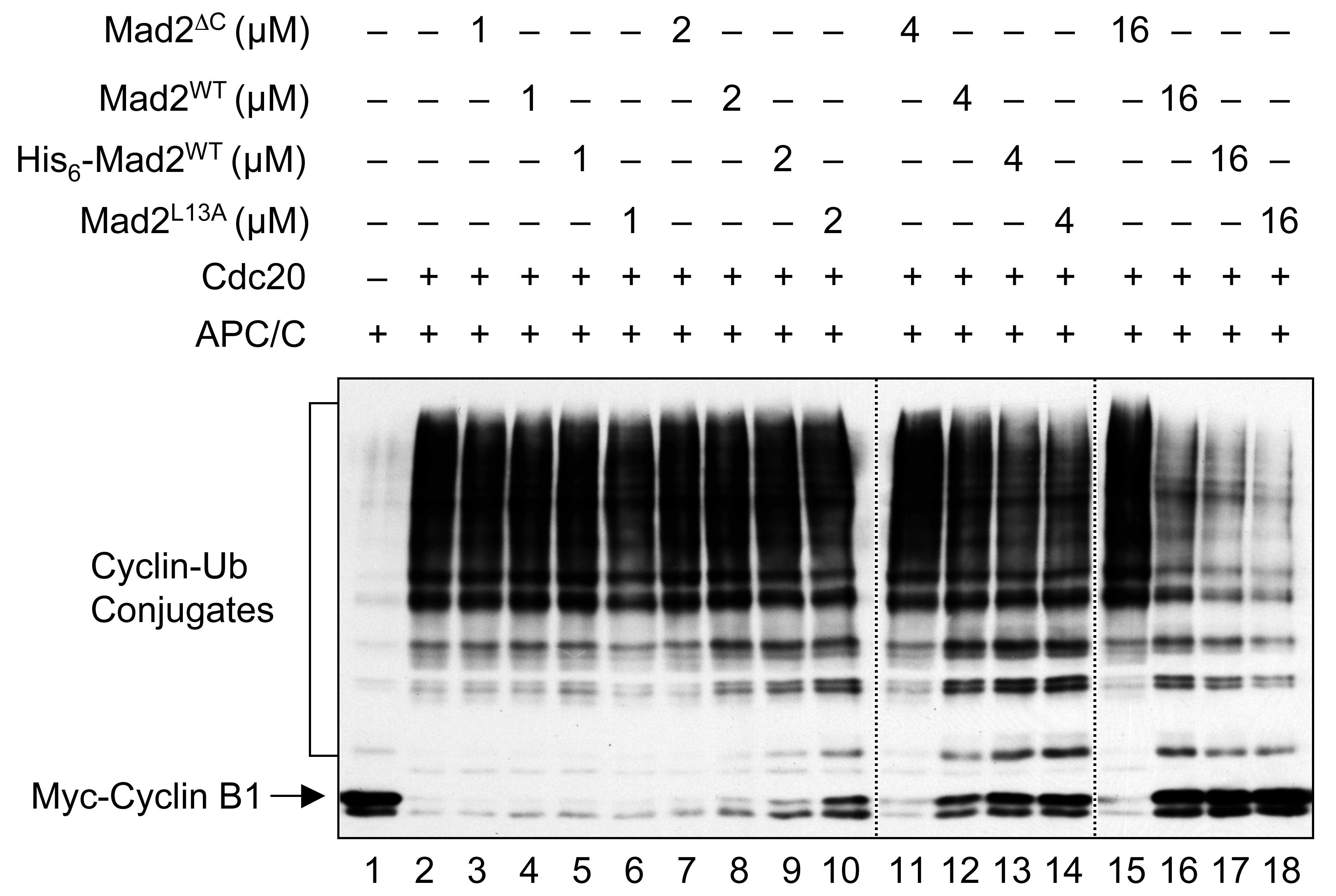

Supplement: Figure S4 — Human Cdc20 was incubated with Mad2WT or Mad2L13A dimers at varying concentrations (1–16 μM) for 2 h. The mixture was then added to APC/C immunopurified from Xenopus egg extracts on anti-APC3 beads for another 1 h. The APC/C beads were then washed and assayed for their ubiquitin ligase activity towards Myc-cyclin B1. The reactions mixtures were blotted with anti-Myc. The unmodified and ubiquitin-conjugated cyclin B1 proteins are indicated. (1.4 MB TIF) [file pbio.0060050.sg004.tif]

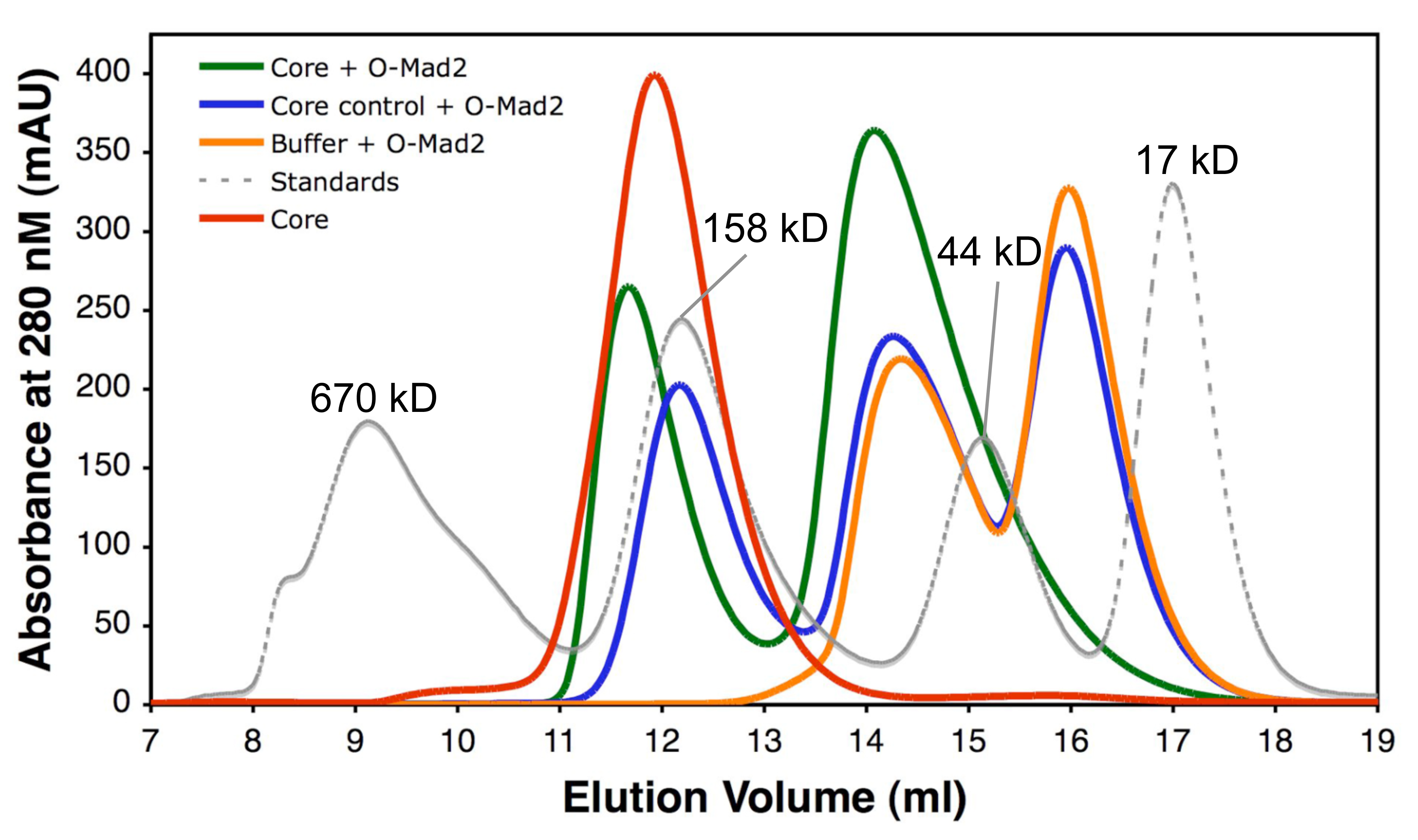

Supplement: Figure S5 — Overlay of the gel filtration chromatograms shown in Figure 7. “Core + O-Mad2” (green): the protein mixture containing O-Mad2 and the Mad1–Mad2 core complex incubated at 37 °C for 30 min; “Core control + O-Mad2” (blue): the protein mixture containing O-Mad2 and the Mad1–Mad2R133E,Q134A core complex incubated at 37 °C for 30 min; “Buffer + O-Mad2” (orange): O-Mad2 incubated with buffer at 37 °C for 30 min; “Standards” (gray): molecular weight standards are shown with a dashed gray line with the native molecular mass of each standard indicated; and “Core” (red): the Mad1–Mad2 core complex alone. (3 MB TIF) [file pbio.0060050.sg005.tif]

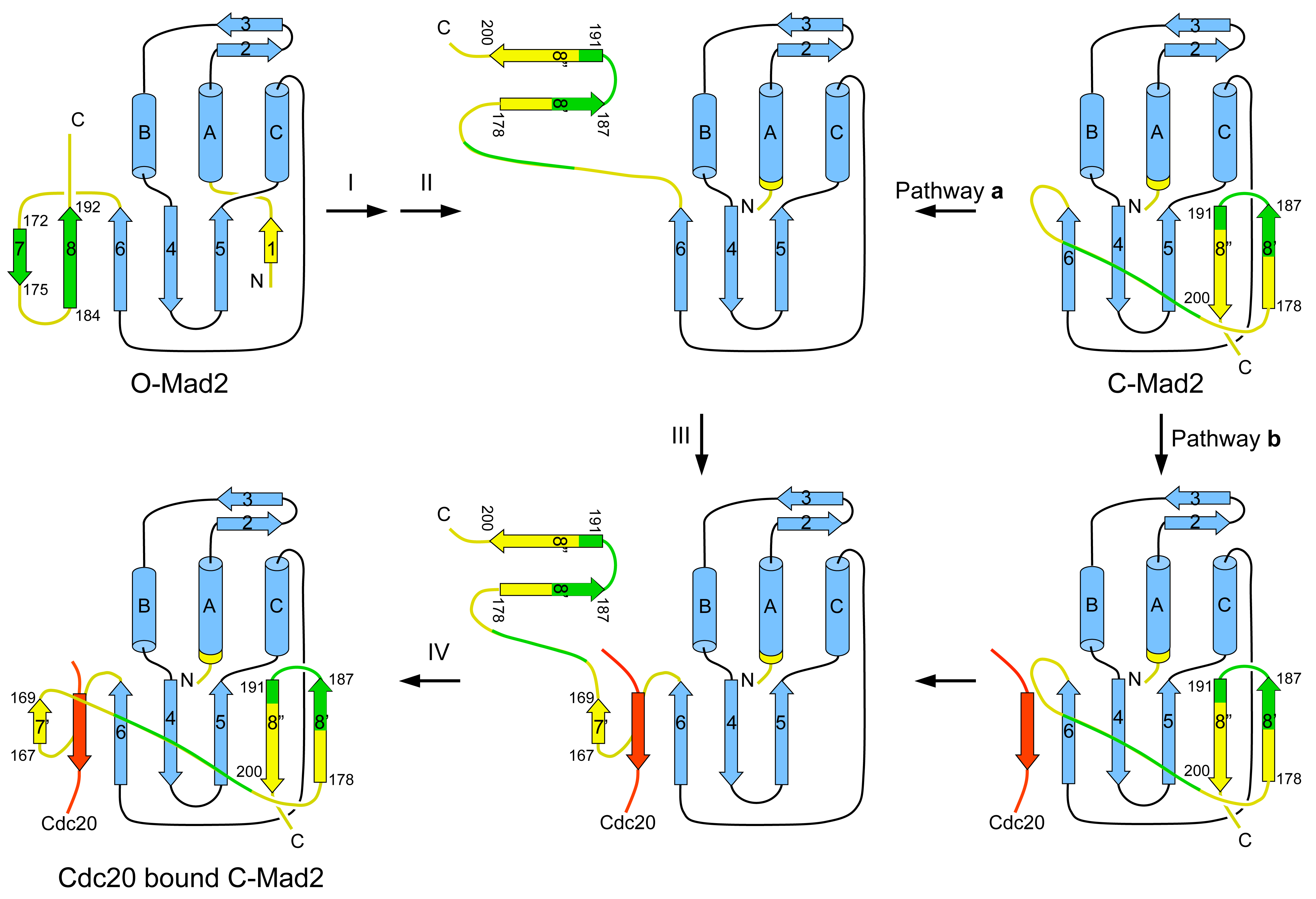

Supplement: Figure S6 — Topology diagrams that illustrate the structural changes in different Mad2 species and the proposed pathways for their binding to Cdc20 are shown. The secondary structural elements are labeled. The core domain for Mad2 is colored blue. The N- and C-terminal regions involved in the Mad2 conformational change are colored yellow, except for residues 172–175 and 184–192, which are shown in green. Cdc20 is shown in red. (694 kB TIF) [file pbio.0060050.sg006.tif]

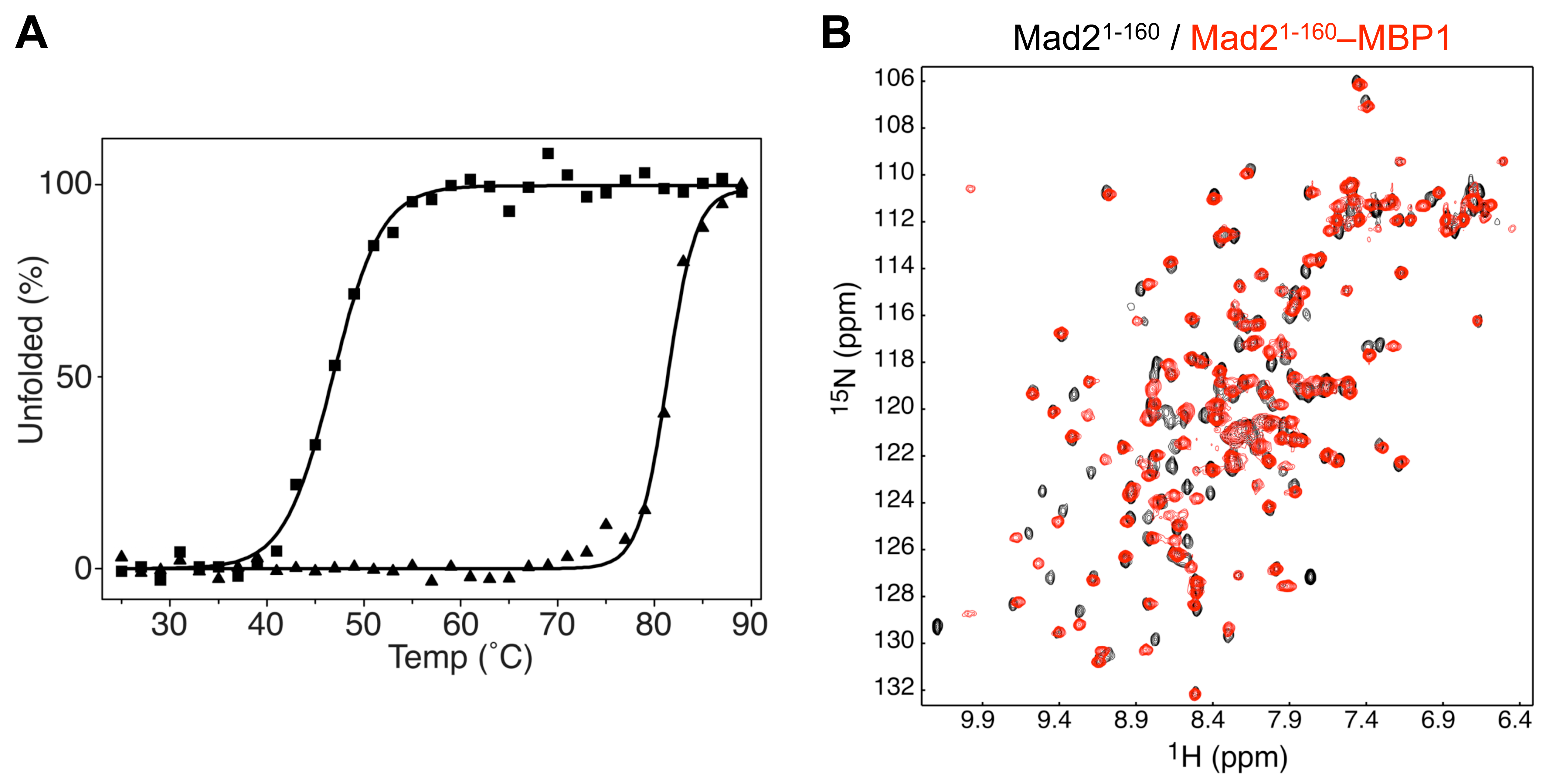

Supplement: Figure S7 — (A) The temperature-induced denaturation curves of Mad2ΔN10 (shown as solid triangles) and Mad21–160 (solid squares) as monitored by the intensity of the circular dichroism (CD) signal at 220 nm. (B) Overlay of 1H-15N HSQC spectra of free Mad21–160 (black) and Mad21–160 in complex with MBP1 (red). The peaks in the Mad21–160 spectra are well dispersed, indicating that Mad21–160 is folded. Several peaks undergo significant chemical shift changes upon the addition of MBP1, indicating that Mad21–160 binds to MBP1. (1.9 MB TIF) [file pbio.0060050.sg007.tif]
